# Teaching style and academic engagement in pre-service teachers during the COVID-19 lockdown: Mediation of motivational climate

**DOI:** 10.3389/fpsyg.2022.992665

**Published:** 2022-10-13

**Authors:** Ginés D. López-García, María Carrasco-Poyatos, Rafael Burgueño, Antonio Granero-Gallegos

**Affiliations:** ^1^Department of Education, Faculty of Education Sciences, University of Almería, Almería, Spain; ^2^Health Research Center, University of Almería, Almería, Spain

**Keywords:** autonomy support, controlling style, mastery motivational climate, performance motivational climate, resilience

## Abstract

COVID-19 and the resulting confinement has had a great impact on the educational environment. Although research in initial teacher education has focused on studying the factors that lead to increased academic engagement, there is no evidence that examines the role of teacher interpersonal style and motivational classroom in a virtual learning context. The aim of this research was to analyze the mediating role of motivational climate between teacher interpersonal style (i.e., autonomy support and controlling style) and academic engagement climate in pre-service teachers in a resilient context. The research design was observational, descriptive, cross-sectional, and non-randomized. A total of 1,410 university students (pre-service teachers) participated (*M*_*age*_ = 23.85; *SD* = 5.13) (59.6% female; 40.3% male; 0.1% other). The scales of interpersonal teaching style, classroom motivational climate, academic commitment, and resilience were used, and a structural equation analysis with latent variables was carried out controlling resilience and gender. The results of the structural equation model show the importance of student perception of teacher autonomy support on academic engagement of the pre-service teachers, as well as the mediation of the mastery climate between autonomy support and academic engagement. For this reason, in order to increase academic engagement in pre-service teachers, it is recommended the use of motivating styles and instructional strategies focused on supporting the decision-making process, initiative, and significant learning.

## Introduction

The situation caused by the COVID-19 pandemic has meant a change of approach to the teaching-learning process at all educational levels, including in initial teacher training (Camargo et al., 2020). During the first phase of the pandemic, characterized by a period of confinement, initial teacher training programs had to adapt quickly and abruptly to the new training reality, which meant moving from a face-to-face training model to a virtual one (Lorenzo-Lledó et al., 2021). For pre-service teachers, this new scenario meant facing both the inconveniences related to a virtual educational model (e.g., lack of digital competence, lack of technological resources, problems with the Wi-Fi connection, or lack of planning), and with their home conditions (e.g., sharing spaces with other family members, stress, or difficulties in concentrating), which led to them experiencing low engagement levels toward their initial training program (Scull et al., 2020).

Academic engagement has been identified as the cornerstone of all educational outcomes as it is closely linked to the quality of the training process for every student, including pre-service teachers (OECD, 2014). Indeed, it is thought that academic engagement could be influenced to a greater extent by the role that the teacher trainer, as a teacher, might adopt within the classroom. According to the Achievement Goal Theory (AGT; Ames, 1992), the teacher’s role is recognized as being amongst the environmental factors that can influence different variables at the academic level (e.g., academic engagement) and that the motivational climate generated in the classroom by the teacher is so important that some authors emphasize it may be responsible for the academic success or failure of students (Ntoumanis and Biddle, 1999). Previous studies (Milton et al., 2018; Mastagli et al., 2021) have shown the need to combine Self-Determination Theory (SDT; Ryan and Deci, 2017) with the AGT (Ames, 1992) when delving into the influence of the classroom social environment, for example, taking into account the interpersonal teaching style. However, to date, no recorded studies have examined the effects of the classroom social environment generated by the teacher trainer on the academic engagement of pre-service teachers during confinement, a period characterized by a virtual training model and a resilient environment (i.e., one that favors both the capacity for recovery in the face of adversity and for adapting to changing demands; Tugade and Fredrickson, 2004). Therefore, this research aims to analyze the potential role that social and environmental factors in the classroom (i.e., interpersonal teaching style and motivational climate) might play in favoring academic engagement in future teachers within a resilient context, acquiring an important role in the context provoked by the COVID-19 pandemic.

### Academic engagement

Academic engagement has been conceptualized as the positive affective and mental state related to academic work, characterized by high levels of energy, enthusiasm, and immersion in activities in which time goes by unnoticed (Schaufeli et al., 2006). The previous research (Schaufeli et al., 2006) operationalized academic engagement through the dimensions of vigor (the perception of high energy levels during study), dedication (the perception of high involvement in studies) and absorption (the perception of high levels of immersion and concentration presented by any academic task). Previous studies have shown the positive relationship between academic engagement and a number of adaptive educational consequences, such as persistence during the teacher training program (Fokkens-Bruinsma and Canrinus, 2015), teacher effectiveness (Kim and Corcoran, 2018) and the intention to complete the training program (Rots et al., 2014), amongst teachers in initial training. Given the importance of academic engagement to the training process of the future, it is necessary to delve into those factors that might determine it in the context of initial teacher training. Specifically, previous research has shown that academic engagement could be greatly influenced by an environment characterized by resilience (Ojo et al., 2021; Wang et al., 2021; Zhang et al., 2021), as well as by prior motivational experiences (Howard et al., 2021). Accordingly, the possible role of the motivational climate in the classroom is considered a proximal factor of teachers’ academic engagement in initial training (OECD, 2014).

### Motivational climate

One of the theories that can explain the motivational aspects related to academic engagement is the AGT (Ames, 1992). AGT is a verified theory in the educational field. It states that, in an achievement-related situation, a person’s motivation and associated behaviors are affected by how success is perceived, and how competence is evaluated. Motivational climates indicate how the different goal factors of the educational context influence achievement in the school environment (Alonso-Tapia et al., 2019). According to the AGT, there are two predominant motivational climates in social contexts of achievement situations: the mastery climate (MC) and the performance climate (PC) (Walling and Duda, 2016). The MC refers to that focused on cooperation between students, where each plays a role in the class and success is based both on the learning process and on intrapersonal criteria related to effort and personal improvement. The PC favors success based on normative and interpersonal criteria, where a punitive response to errors is found and where there is rivalry between students (Ames, 1992). According to the existing literature, the motivational climate in the classroom can affect the adaptive patterns of students (Madjar et al., 2019). Conversely, PC is negatively related to variables such as perceived competence (Granero-Gallegos et al., 2021) and academic performance (Gutiérrez and Tomás, 2018). Various studies have shown that a positive MC is associated with better cognitive and motivational outcomes, including academic engagement (Patrick et al., 2011; Reyes et al., 2012). Research has also suggested that the motivational climate in the classroom might be partly due to the type of teaching style used by the teacher trainer (Granero-Gallegos et al., 2021).

### Interpersonal teaching style

SDT understands interpersonal style to be the way in which the teacher trainer interacts, communicates, and relates to teachers in initial training during the teacher-training program (Ryan and Deci, 2020; Ryan et al., 2021). SDT differentiates at least two types of interpersonal styles—a support for autonomy style, referring to those teaching strategies that provide the student with opportunities to choose and make decisions, giving reasoned explanations for tasks, recognizing the opinions of students and identifying their interests (Ryan and Deci, 2020; Ryan et al., 2021), and a controlling style, referring to those teaching strategies that pressure students into thinking, behaving and feeling in a way that is pre-established by the teacher, where the teacher imposes his/her own agenda and the requirements to be met by the student, regardless of the students’ interests (Ryan and Deci, 2020; Ryan et al., 2021).

SDT-based research has shown that the autonomy support style and the controlling style are two independents, yet related, variables (Reeve et al., 2014; Ryan and Deci, 2017; Opdenakker, 2021). This implies that both interpersonal styles can coexist in the same context and contribute in a differentiated way to the prediction of specific consequences. Specifically, it is argued that the autonomy support style would not only favor positive consequences but would also dampen negative experiences. Conversely, it is believed that the controlling style would facilitate maladaptive consequences and, at the same time, undermine positive experiences (Opdenakker, 2021). Previous studies with university students have observed that the autonomy support style was positively related to both MC (Granero-Gallegos et al., 2021) and to academic engagement (Jiang and Zhang, 2021; Ma, 2021). In contrast, the controlling style was positively associated with maladaptive consequences, such as PC (Moreno-Murcia et al., 2018), while it was negatively related to adaptive consequences (Moreno-Murcia et al., 2018).

### The present study

**FIGURE 1:**
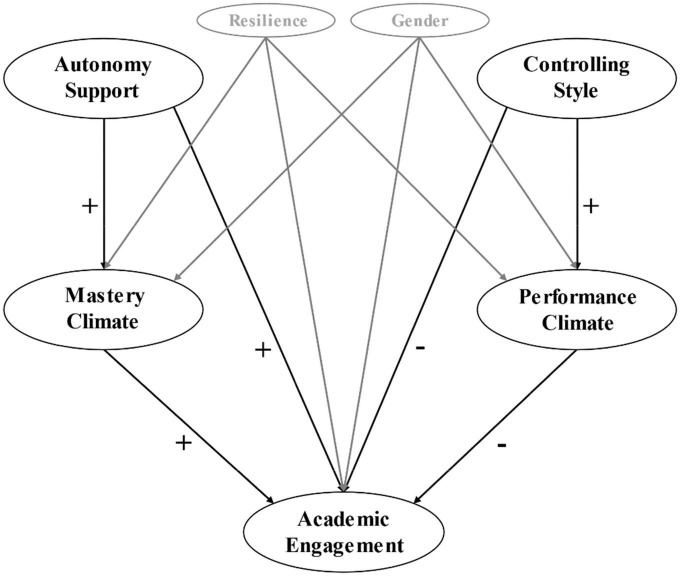
Predictive relationships of the interpersonal teaching style on academic engagement through the mediating role of the motivational climate, controlling the gender and resilience variables.

Despite the importance attributed to academic engagement as a cornerstone of the pre-service teacher training process, little is known about the role that interpersonal style and the motivational climate generated by the teacher trainer might play in the academic engagement of pre-service teachers. To date, no scientific evidence has been found that studies this relationship while also considering the resilience of pre-service teachers in terms of the motivational and cognitive abilities provoked by the pandemic environment caused by COVID-19. This aspect is also significant, and an important contribution to the scientific literature given the scarcity of studies looking at the influence of interpersonal teaching styles on motivational and cognitive variables affecting pre-service teachers in the pandemic period. Therefore, the objective of this study is to analyze the mediating role of the motivational climate between the interpersonal teaching style and academic engagement in trainee teachers in a resilient context. The following hypotheses were established: First, that the perception of autonomy support will be positively related to academic engagement (H1); second, that the controlling style will be negatively related to academic engagement (H2); third, that the MC will be positively associated with academic engagement (H3): fourth, that the PC will be negatively associated with academic engagement (H4); fifth, that the MC acts as a positive mediator between autonomy support and academic engagement (H5); and sixth, that the PC acts as a negative mediator between the controlling style and academic engagement (H6); (Figure 1). The *Strengthening the Reporting of Observational Studies in Epidemiology* (STROBE) *Initiative* (Von Elm et al., 2007) was used for the study description.

## Materials and methods

### Design

The research design was observational, descriptive, cross-sectional, and non-randomized. The sample was made up of university students from several Andalusian universities. The data were collected at the end of the 2020/2021 academic year. The following inclusion criteria were established: (i) to be a student of the Master’s Degree in Secondary and Upper-Secondary Education Teaching, Vocational Training and Language Teaching or a student of a University Degree (face-to-face study) dedicated to the initial training of teachers. The exclusion criteria were: (i) not consenting to the use of data in the study; (ii) not completely filling in the data collection form.

### Instruments

#### Interpersonal teaching style in higher education (EIDES)

The Spanish version by Granero-Gallegos et al. (2021) was used, comprising 11 items that measure the students’ perception of the teacher’s controlling style (6 items) (e.g., “My teacher has paid less attention to students he/she disliked”) and support for teacher autonomy (5 items) (e.g., “My teacher has offered different opportunities and options during the class”). The responses were collected on a Likert scale ranging from 1 (*completely disagree*) to 5 (*completely agree*).

#### Motivational climate in higher education (MCES)

The Spanish version by Granero-Gallegos and Carrasco-Poyatos (2020) was used. The instrument comprises a total of seven items that make up two factors measuring the motivational climate of the classroom: the mastery climate (4 items) and the performance climate (3 items). The responses were collected on a Likert scale ranging from 1 (*completely disagree*) to 5 (*completely agree*).

#### Academic engagement

The Spanish version of the *Utrecht Work Engagement Student Scale* (UWES-SS) (Schaufeli et al., 2002) was used. The instrument consists of 17 items that make up three factors: vigor (6 items) (e.g., “In my studies I feel full of energy”), dedication (5 items) (e.g., “My studies are stimulating and inspiring”) and absorption (6 items) (e.g., “I am immersed and focused on my studies”). The responses were collected on a Likert scale ranging from 1 (*completely disagree*) to 5 (*completely agree*). Academic engagement was calculated as the average value of the scores for each of the factors that comprise it.

#### Resilience

The reduced version by Notario-Pacheco et al. (2011), adapted to the Spanish university context, was used. This instrument comprises 10 items (e.g., I can adapt to changes) that are organized in a dimension that measures resilience in young adults. The responses were collected on a Likert scale ranging from 0 (*never*) to 4 (*always*). High scores on the scale indicate a high level of resilience.

### Procedure

Firstly, the heads and teachers of the Faculties of Education Sciences and the Master’s Degree in Secondary and Upper-Secondary Education Teaching, Vocational Training and Language Teaching were contacted to request permission and to ask for their collaboration in the research. The researchers then contacted the students by email. The data were collected using an online form throughout May 2021. The form briefly explained the importance of the research, the anonymity of the responses, the way to complete the scale, that the responses given would not affect any qualification in any way, and that the participants could stop participating in the study at any time. All participants gave their consent to be included in the study prior to participating. The research was conducted in accordance with the Declaration of Helsinki and the protocol was approved by the Bioethics Committee of the University of Almería (Ref: UALBIO2021/009).

### Risk of bias

Regarding the risk of bias, it should be noted that there was no sample randomization since convenience sampling was employed. However, there was blinding between the participants and the researchers in charge of data treatment and analysis. With respect to selection bias, participation was voluntary and communication with participants was conducted by email.

### Sample size

An *a priori* analysis was carried out on the statistical power of the adequate sample size for meeting the study objective. Using the *Free Statistics Calculator* v.4.0 software (Soper, 2022), it was estimated that a minimum of 1,401 participants were needed for f^2^ = 0.15 effect sizes with a statistical power of 0.99 and a significance level of α = 0.05 in a structural equation model with six latent variables and 32 observable variables. In the actual study, 1,410 university students took part.

### Data analysis

The descriptive statistics of each factor were calculated as well as the asymmetry, kurtosis, Cronbach’s alpha (α), and the correlation between dimensions, using SPSS v.27 software. The hypothesized predictive relationships of the interpersonal teaching style on academic engagement, mediated by the motivational climate, were verified with a structural equations model (SEM) of the latent variables using AMOS v.25. Following the proposal of Wang et al. (2017), model analysis was performed using the two-step method. In the first step, the saturated model was examined by relating all the dimensions to each other. In the second step, the predictive relationships of the hypothesized model were evaluated. The evaluation of the models was performed taking into account the following goodness-of-fit indices: the values of the χ^2^/gl ratio, CFI (*Comparative Fit Index*), TLI (*Tucker–Lewis Index*), RMSEA (*Root Mean Square Error of Approximation*) with its 90% confidence interval (CI), and SRMR (*Standardized Root Mean Square Residual*). For the χ^2^/gl ratio, values < 5.0 are considered acceptable (Hu and Bentler, 1999); CFI and TLI values between 0.90 and 0.95, and RMSEA and SRMR values < 0.08, are considered to have acceptable goodness-of-fit indices (Marsh et al., 2004; Hooper et al., 2008). In addition, RMSEA values < 0.06 and SRMR values < 0.05 are considered to have excellent model goodness-of-fit indices (Hu and Bentler, 1999; Hooper et al., 2008). The internal consistency of each scale was evaluated with different parameters: α, composite reliability (CR), H coefficient, and AVE (*Average Variance Extracted*) to measure convergent validity. Reliability values ≥ 0.70 and AVE values > 0.50 are considered acceptable (Dominguez-Lara, 2017; Hair et al., 2018).

In the hypothesized model, the following direct relationships were established: between the dimensions of the interpersonal teaching style and the two dimensions of motivational climate and academic engagement; and between MC, PC and academic engagement. Indirect relationships were established between autonomy support, controlling style and academic engagement through the MC and PC. As the Mardia coefficient values were high (> 123.67; *p* < 0.001), the analyses were performed using the maximum likelihood estimation method and the 5,000-iteration *bootstrapping* procedure (Kline, 2016). In addition, R^2^ was used for the effect sizes (ES) in order to improve the results interpretation, since it estimates the degree of influence by quantifying the variance percentage of the dependent variable explained by the predictors (Dominguez-Lara, 2017). The cut-off points were: 02, 0.13, and 0.26, for small, medium, and large effect sizes, respectively (Cohen, 1992). Furthermore, the confidence intervals (CI: 95%) were calculated to ensure that no R^2^ value was < 0.02, as this is the minimum required for the interpretation.

## Results

### Participants

A total of 1,410 university students participated (841 women, 568 men, 1 other) from the Master’s Degree in Secondary and Upper-Secondary Education Teaching, Vocational Training and Language Teaching from eight Andalusian public universities (Spain). The age of the participants was between 21 and 60 years (*M* = 23.85; *SD* = 5.13). There were no missing values in the included sample data.

### Preliminary analyses

The descriptive statistics and the correlations between the study’s latent variables are presented in Table 1.

**TABLE 1:** Descriptive statistics and correlation between variables.

Variable	*M*	*SD*	Q1	Q2	α	CR	AVE	H	2	3	4	5	6
1. Autonomy Support	3.65	0.83	–0.38	−0.25	0.85	0.82	0.53	0.83	–0.36**	0.38**	0.79**	–0.17	0.56**
2. Controlling Style	2.30	0.95	0.44	−0.51	0.88	0.88	0.54	0.88		−0.15**	−0.31**	0.71**	–0.07
3. Resilience	4.09	0.68	–0.40	−0.62	0.09	0.89	0.52	0.90			0.32**	–0.04	0.37**
4. Mastery Climate	3.88	0.76	–0.51	−0.01	0.78	0.79	0.51	0.82				–0.17	0.59**
5. Performance Climate	2.47	0.97	0.45	−0.29	0.72	0.74	0.51	0.84					–0.02
6. Academic Engagement	3.51	0.82	–0.31	−0.07	0.90	0.89	0.54	0.91					

**The correlation is significant at the 0.01 level. *M*, mean; *SD*, standard deviation; Q1, skewness; Q2, Kurtosis; α, Cronbach’s alpha; CR, composite reliability; AVE, average mean extracted; *H*, H coefficient.

### Main analyses

In Step 1, the model presented acceptable goodness-of-fit indices: χ^2^/gl = 2.662, *p* < 0.001; CFI = 0.944; TLI = 0.937; RMSEA = 0.043 (90%CI = 0.040;0.045; *p_*close*_* = 1,000), SRMR = 0.049. In Step 2, the predictive SEM model showed the following acceptable goodness-of-fit index: χ^2^/gl = 3.470, *p* < 0.001; CFI = 0.945; TLI = 0.938; RMSEA = 0.042 (90%CI = 0.040;0.044; *p_*close*_* = 1,000), SRMR = 0.047. The explained variance was 63% for the MC, 51% for the PC, and 44% for academic engagement. In the SEM model, after controlling for gender and resilience, the direct relationships between the perception of a controlling style by the teacher and the MC, as well as the direct relationship between the PC and academic engagement, were not significant; nor were the direct effects of resilience on the two dimensions of motivational climate and PC on academic engagement. In contrast, the direct relationships between the perception of autonomy support and MC, and between PC and academic engagement, were statistically significant, as were the direct effects of the controlling style, the PC and academic engagement. Finally, the direct relationship between resilience and academic engagement was also statistically significant, as was the gender variable, which showed that, among women, the relationship between the prediction and academic engagement was higher.

**FIGURE 2:**
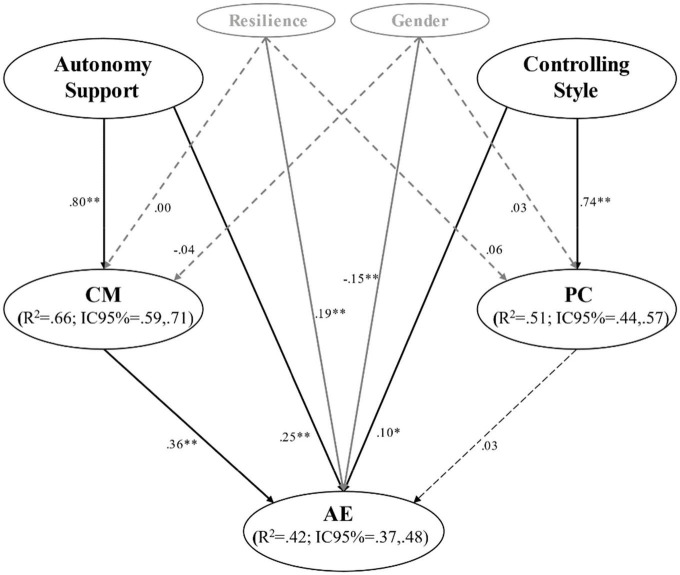
Predictive relationships of the interpersonal teaching style on academic engagement through the mediating role of the motivational climate, controlling the gender and resilience variables. **p < 0.001. AE, Academic engagement; MC, Motivational climate; PC, Performance climate; R^2^, Explained variance; CI, Confidence interval. The dashed lines represent non-significant relationships.

Regarding the effects of mediation, the MC was a mediator between the teacher autonomy support style and academic engagement (β = 0.29; *p* < 0.001). However, the PC did not act as a mediator between any of the interpersonal teaching style factors and academic engagement. With regard to the total effects, it is noteworthy that the prediction of the perception of autonomy support on academic engagement, mediated by the MC, supposes an increase in the predictive relationship (β = 0.54; *p* < 0.001) between the above two variables. Figure 2 shows the CI (95%) of R^2^, which can be considered as ES measures (Dominguez-Lara, 2017) and, in all cases, this is large.

## Discussion

The objective of this research was to analyze the relationships of both the interpersonal teaching style of autonomy support and the controlling style on academic engagement in a sample of teachers in initial training, examining the mediating role of the motivational climate in the resilient context caused by the pandemic. The main results highlight the importance of the mediation of MC between the autonomy support style and academic engagement.

In accordance with the posited hypotheses, the results reveal that, during the training process, the autonomy support style had a positive direct effect on the academic engagement of future teachers (H1) as well as a positive indirect effect mediated by the MC (H5). Although both relationships were statistically significant, it should be noted that the magnitude of the effect size was greater in the relationship between autonomy support and academic engagement mediated by the MC. These results corroborate previous studies that showed the importance of motivational climate mediation on mastery between the teaching styles and the cognitive (Jiang and Zhang, 2021) and behavioral (Granero-Gallegos et al., 2021) consequences in the university environment. In this way, teacher trainers who support student autonomy will generate a motivational climate oriented toward a process that will develop greater learning engagement from teachers in initial training. This might be because MC is a positive predictor of more self-determined motivational styles, regulating the students’ behavior to incorporate learning-directed skills (Standage et al., 2003; Kipp and Amorose, 2008; Hodge and Gucciardi, 2015). In fact, using autonomy-supporting teaching styles tends to motivate students toward the self-realization of their goals and making it more likely that they develop higher levels of interest (Ryan and Aikenhead, 1992); that is to say, support for student autonomy has an effect on intrinsic motivation, resulting in students being more interested in the task they are performing (Reeve, 2009). Therefore, establishing motivational climates that foster interpersonal relationships in the classroom leads to students adopting a self-regulated approach to the work they are undertaking and results in increased academic engagement (Carmichael et al., 2017).

In addition to examining the indirect relationship of autonomy support to academic engagement, the present research also showed the positive direct relationship of MC on academic engagement (H3). These results are in line with previous studies, such as those by Sevil Serrano et al. (2016) and Gutiérrez and Tomás (2018), underlining the importance of generating a process-focused socio-contextual climate, which can contribute to improving learning outcomes in initial teacher training. This may be due to the importance of the educational context in shaping the students’ academic engagement (Wang and Eccles, 2013). Specifically, the MC regulates the behavior patterns of students to incorporate skills directed toward the learning process (Hodge and Gucciardi, 2015). Therefore, the future teachers will perceive a social environment in the classroom particularized by participation, effort, cooperation, and improvement when undertaking learning tasks and thus they will feel academically involved in the training process.

The findings reveal that when a controlling style is perceived, it has a direct positive effect on the academic engagement of future teachers, thus falsifying H2. The present research also showed the absence of a relationship between the PC and academic commitment, discounting H4 and H6. Although the PC does not act as a mediator between the controlling teaching style and academic engagement (H6), the direct relationship between the controlling style and the PC is statistically significant and positive, thus meeting H7. However, perceiving a controlling style on the part of the teacher can generate a positive predictive relationship to academic engagement, although less than the predictive relationship of perceiving an autonomy support style. These results do not support the findings of previous research, such as those of Soenens et al. (2012) and De Meyer et al. (2014), in showing a negative relationship between the controlling teaching style and student engagement. This contradictory result could be due to the traditional use in classrooms of a controlling teaching style by teachers in the academic field, as expressed by authors such as Assor et al. (2002), which may mean that some students are used to being directed and need to feel controlled during the learning process in order to advance.

Following the hypothesized model, the findings of the present research contribute to the scientific evidence on the relationship between resilience and academic engagement. Previous studies, such as Medina et al. (2020) or Koob et al. (2021) corroborate the significant and positive relationship between resilience and academic engagement in a virtual training context caused by the COVID-19 pandemic. These results may be due to the protective role that resilience plays on the uncertainty factors caused by COVID-19 in students (Gundogan, 2021; Herbers et al., 2021). Thus, trainee teachers who have a greater capacity to overcome adverse situations will experience greater study engagement. In addition, the results of the present study demonstrate a greater predictive relationship to academic engagement among girls than among boys. This result corroborates the findings of several previous works in this field (Driessen and van Langen, 2013; Kim and Corcoran, 2017). Studies such as those of Tison et al. (2011) and Kessels et al. (2014) demonstrate higher average values of academic engagement in women than in men. One possible explanation may be due to the important role of gender identity, socially created for teaching careers (Kessels et al., 2014). As a result, female teachers in training will be more academically engaged in their studies.

Finally, the educational relevance of the MC should be highlighted. This is generated through the interpersonal teaching style of autonomy support in a context of resilience, resulting in greater academic engagement in teachers undergoing training.

### Limitations and future prospects

Despite the above findings, the present research also has certain limitations. First, the convenience sampling method used means the results obtained should be interpreted with caution. Second, no experimental intervention was established with different teaching styles to allow us to verify their effects on the students’ perceptions. As future lines of research, it would be important to establish intervention protocols that measure the effect of different teaching styles on the academic engagement of trainee teachers by creating both motivational classroom climates. Finally, the measurement of teaching intervention styles and motivational classroom climates was only carried out *via* questionnaires. Therefore, it is recommended that future lines of research use complementary observational instruments to triangulate the data obtained.

### Practical implications for initial teacher training

The results of this study suggest the need to use motivating styles characterized by autonomy support, and to generate mastery climates that foster academic engagement in future teachers. In this regard, teacher trainers need to use instructional strategies focused on providing meaningful choice, initiative, and justification: (a) providing coherent explanations on course objectives, content, homework, and other learning tasks. (b) Using cooperative learning strategies based on group training in which students can work cooperatively by discussing ideas, providing feedback, and sharing the necessary resources with the rest of their classmates. (c) Establishing opportunities of choice to learn and develop knowledge and to perform learning tasks around their preferences and interests. (d) Establishing assessment processes that promote student feedback in the teaching process.

## Data availability statement

The raw data supporting the conclusions of this article will be made available by the authors, without undue reservation.

## Ethics statement

The studies involving human participants were reviewed and approved by University of Almería (Ref: UALBIO2021/009). The patients/participants provided their written informed consent to participate in this study.

## Author contributions

AG-G, GL-G, and RB conceived the hypothesis of this study. AG-G, GL-G, and MC-P participated in data collection. RB and AG-G analyzed the data. GL-G, AG-G, RB, and MC-P wrote the manuscript with the most significant input from AG-G. All authors contributed to the data interpretation of statistical analysis and read and approved the final manuscript.
